# Recurrent mutations of MAPK pathway genes in multiple myeloma but not in amyloid light-chain amyloidosis

**DOI:** 10.18632/oncotarget.12029

**Published:** 2016-09-15

**Authors:** Seok Jin Kim, Hyun-Tae Shin, Hae-Ock Lee, Nayoung K.D. Kim, Jae Won Yun, Jee Hyang Hwang, Kihyun Kim, Woong-Yang Park

**Affiliations:** ^1^ Division of Hematology-Oncology, Department of Medicine, Samsung Medical Center, Sungkyunkwan University School of Medicine, Seoul, Korea; ^2^ Samsung Genome Institute, Samsung Medical Center, Department of Molecular Cell Biology, Sungkyunkwan University School of Medicine, Seoul, Korea; ^3^ Samsung Biomedical Research Institute, Samsung Medical Center, Seoul, Korea

**Keywords:** multiple myeloma, cancer panel, MAPK pathway, amyloidosis

## Abstract

Clinically applicable platforms revealing actionable genomic alterations may improve the treatment efficacy of myeloma patients. In this pilot study, we used a high depth targeted sequencing panel containing 83 anti-cancer drug target genes to sequence genomic DNAs extracted from bone marrow aspirates of 23 patients with myeloma and 12 patients with amyloid light-chain (AL) amyloidosis. Mutation analysis revealed NRAS as the most commonly mutated gene (30%, 7/23) in myeloma patients followed by KRAS (26%, 6/23) and BRAF (22%, 5/23). However, no significant mutations were found in the 12 patients with AL amyloidosis. Notably, 6 of the 23 myeloma patients showed multi-site and/or multi-gene mutations in NRAS, KRAS, or BRAF, indicating compound aberrations in the Mitogen activated protein kinase (MAPK) pathway. Gene panel sequencing also revealed cytogenetic abnormalities associated with prognosis in myeloma patients. In conclusion, our pilot study suggests that targeted gene sequencing may have an important prognostic value for myeloma patients for the identification of actionable genomic alterations and cytogenetic aberrations.

## INTRODUCTION

Since novel drugs such as proteasome inhibitors and immunomodulatory agents were first introduced for the treatment of multiple myeloma, survival outcomes have significantly improved [1–3]. However, multiple myeloma still remains an incurable disorder because almost all patients relapse and become refractory to salvage treatments [4, 5]. As treatment outcome may be associated with cytogenetic abnormalities at diagnosis, patients with multiple genetic alterations might show worse prognosis than patients without them [6, 7]. Genetic alterations may accumulate during the development of myeloma from asymptomatic monoclonal gammopathy of undetermined significance because myeloma cases are preceded by an asymptomatic expansion of clonal plasma cells [8, 9]. Amyloid light-chain (AL) amyloidosis is a rare clonal plasma cell disorder characterized by deposition of amyloid fibrils derived from immunoglobulin light chains in various organs [10]. AL amyloidosis and multiple myeloma are theoretically same disease entity at the cellular level, and AL amyloidosis was reported to share genetic susceptibility with multiple myeloma [11]. The presence of small plasma cell clones in AL amyloidosis implies that these clones could become larger, leading to overt multiple myeloma over time [12]. Thus, patients with myeloma may have heterogeneous subclones, and the identification of genetic abnormalities with low frequency in these subclones could be helpful for a better understanding of each patient with myeloma. However, it is difficult to detect low frequency genetic alterations in small subclones by conventional molecular testing using malignant plasma cells from bone marrow. Genomic next generation sequencing (NGS) has allowed for a thorough exploration of the genetic alterations possible in myeloma [13, 14]. However, NGS is still not readily used in clinical practice because it is both a high cost and time-consuming process. Thus, the development of clinically applicable platforms revealing genomic alterations could help to improve a risk-adapted treatment strategy. Here, we performed a pilot study to evaluate the feasibility of a targeted gene sequencing panel consisting of 83 genes in myeloma and AL amyloidosis patients.

## RESULTS

### Characteristics of patients

**Figure 1:**
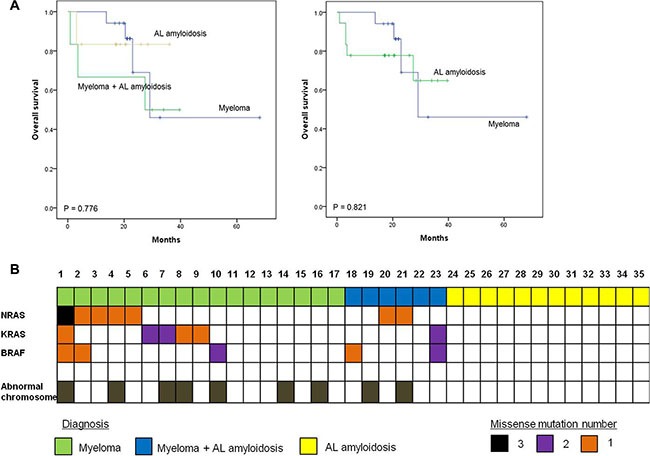
Survival plots for multiple myeloma patients with or without amyloidosis (**A**) Survival outcome of patients in different groups (**B**) *NRAS, KRAS*, and *BRAF* (MAPK pathway) mutation profile in multiple myeloma and AL amyloidosis patients

A total of 35 patients was enrolled in this study; the median age at diagnosis was 60 years (range: 31–85). Patients were classified into one of three groups according to the percentage of plasma cells and the presence of organ amyloidosis and osteolytic lesions: myeloma (*n* = 17), myeloma with AL amyloidosis (*n* = 6), and AL amyloidosis (*n* = 12). Thus, 17 patients who were diagnosed with myeloma had at least 10% malignant plasma cells in their bone marrow in conjunction with increased M protein in the serum and/or urine. With the exception of one patient, all myeloma only patients also had at least one sign or symptom related to myeloma such as renal dysfunction, anemia, or osteolytic bone lesion (Table 1). In the remaining 18 patients, the presence of amyloid depositions in various organs such as heart, kidney and gastrointestinal tract was pathologically confirmed; thus, they were diagnosed with AL amyloidosis. Six of these patients had ≥ 30% plasma cells in their bone marrow aspirates or had osteolytic lesions diagnosed as myeloma coupled with AL amyloidosis (Table 2). The clinical and laboratory characteristics of patients at diagnosis and their initial treatments and transplantations are summarized in Tables 1 and 2. The most commonly used treatment regimens were thalidomide, dexamethasone with or without cyclophosphamide (*n* = 13), and bortezomib, melphalan and prednisone (*n* = 9). Bortezomib-containing treatment was used for patients ineligible for autologous stem cell transplant (ASCT), whereas thalidomide-containing treatment was done for patients eligible for ASCT. Thus, ASCT was done in 13 patients as a part of the induction treatment. At the time of analysis, 26 patients were alive, while nine patients died from myeloma (*n* = 4), AL amyloidosis (*n* = 2), or myeloma with AL amyloidosis (*n* = 3). The overall survival was not significantly different among the three groups (Figure 1A). When patients with AL amyloidosis were grouped together regardless of myeloma, the comparison of overall survival was not different either (Figure 1A).

**Table 1:** Characteristics and outcomes of patients with myeloma

No.	S/A	Type	Osteolytic lesion	Plasmacytoma	Hgb < 10 g/dL	Ca > 11.5 mg/dL	LDH increased	BM PC%	ISS	Induction treatment	Response to induction	ASCT	Survival status	OS (months)
1	M/46	λ	Presence					70	I	VD	PD		Dead	23
2	F/75	IgG, λ	Presence	Presence				90	II	VMP	PD		Alive	21
3	M/68	IgA, λ						60	II	VMP	CR		Alive	18
4	M/74	λ	Presence		Presence	Presence		100	III	VMP	PD		Alive	20
5	M/76	IgG, κ						10	I	None	NA		Alive	17
6	F/48	IgG, κ	Presence	Presence	Presence			40	I	VAD	PR	Single	Alive	68
7	F/31	IgG, κ	Presence	Presence		Presence		35	I	TD	PD	Tandem	Dead	14
8	M/71	IgM, κ	Presence					60	I	VMP	SD		Alive	21
9	M/51	IgG, κ	Presence		Presence			23	II	TD	CR	Single	Dead	20
10	M/42	IgG, λ						50	II	TD	VGPR	Single	Alive	22
11	F/85	IgG, κ			Presence			60	II	VMP	SD		Alive	23
12	M/63	IgM, κ	Presence					50	I	TD	PD	Tandem	Alive	22
13	F/68	κ		Presence				40	I	VMP	CR		Alive	22
14	F/54	IgG, κ			Presence		Presence	16	II	TD	SD	Single	Alive	33
15	F/66	IgG, κ			Presence			50	II	CMP	PD		Alive	21
16	M/77	IgG, λ			Presence			100	III	VMP	VGPR		Alive	20
17	M/43	IgA, κ						30	II	TCD	PD	Tandem	Dead	29

S: sex; A: age; Hgb: hemoglobin; LDH: lactate dehydrogenase; BM: bone marrow; PC: plasma cell; DSS: ISS: International staging system; ASCT: autologous stem cell transplantation; OS: overall survival; VD; bortezomib, dexamethasone; VMP: bortezomib, melphalan, prednisone; VAD: vincristine, adriamycin, dexamethasone; TD: thalidomide, dexamethasone; CMP: carfilzomib, melphalan, prednisone; TCD: thalidomide, cyclophosphamide, dexamethasone; CR: complete response; VGPR: very good partial response; PR: partial response; SD: stable disease; PD: progressive disease.

**Table 2:** Characteristics and outcomes of patients with AL amyloidosis including myeloma with amyloidosis

No.	S/A	Type	Osteolytic lesion	Involved organ	Hgb < 10 g/dL	LDH increased	BM PC%	ISS	Combined with MM	Induction treatment	Response to induction	ASCT	Survival status	OS (months)
18	M/37	κ		Heart, GI tract	Presence		30	II	Yes	TCD	PR	Single	Alive	40
19	M/62	IgG, κ	Presence	Kidney	Presence	Presence	100	II	Yes	None	NA		Dead	1
20	M/57	κ		Liver			30	II	Yes	TCD	CR	Single	Alive	34
21	M/57	κ		Heart			30	II	Yes	MD	PD		Dead	4
22	F/49	κ		Liver			50	II	Yes	TD	CR	Single	Alive	30
23	M/71	IgG, κ	Presence	Heart		Presence	20	III	Yes	MD	CR		Dead	27
24	F/67	IgG, κ		Kidney			20		No	MD	CR		Alive	21
25	F/59	λ		Heart			15		No	VMP	CR		Alive	36
26	F/55	κ		Lung		Presence	15		No	TD	PR	Single	Alive	28
27	M/60	λ		GI tract, Nerve			5		No	None	NA		Alive	26
28	F/47	IgG, λ		Kidney, Heart, Nerve			20		No	MD	PD		Dead	3
29	M/77	IgG, λ		Heart		Presence	15		No	None	NA		Dead	3
30	M/60	κ		Liver, nerve			15		No	TD	PR		Alive	14
31	M/57	λ		Heart, nerve			12		No	TD	SD		Alive	20
32	F/64	IgA, λ		Kidney, Heart, Nerve			15		No	MD	PR		Alive	19
33	M/43	IgG, λ		Kidney			4		No	None	CR	Single	Alive	17
34	M/64	λ		Kidney, Soft tissue			15		No	TD	PR	Single	Alive	17
35	M/77	IgA, λ		Kidney, Heart, Nerve			20		No	VMP	SD		Alive	17

S: sex; A: age; Hgb: hemoglobin; LDH: lactate dehydrogenase; BM: bone marrow; PC: plasma cell; DSS: Durie-Salmon stage; ISS: International staging system; ASCT: autologous stem cell transplantation; OS: overall survival; GI tract: gastrointestinal tract; TCD: thalidomide, cyclophosphamide, dexamethasone; MD: melphalan, dexamethasone; TD: thalidomide, dexamethasone; VMP: bortezomib, melphalan, prednisone; CR: complete response; PR: partial response; SD: stable disease; PD: progressive disease; NA: not applicable; MM: multiple myeloma.

### Mutations in myeloma and AL amyloidosis

**Figure 2:**
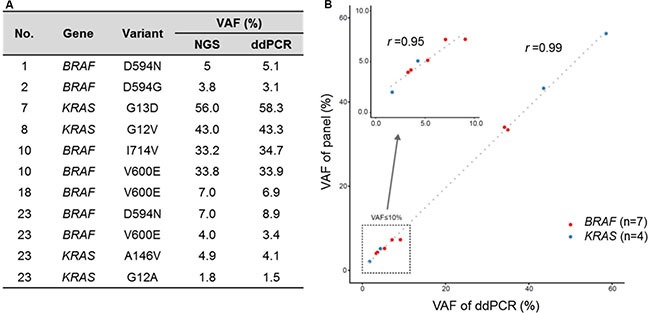
High correlation of VAF from cancer panel and digital droplet PCR (**A**) VAF from cancer panel and ddPCR (**B**) Scatter plot for VAF of cancer panel and ddPCR.

Patients were genotyped using high depth panel sequencing with a mean depth of coverage, 846× (Supplementary Table S1). Mutation analysis of all myeloma patients including the six patients with AL amyloidosis revealed NRAS as the most commonly mutated gene (30%, 7/23), followed by KRAS (26%, 6/23) and BRAF (22%, 5/23) (Figure 1B). One patient (patient #1, Table 3) showed multiple mutations in NRAS, KRAS, and BRAF, and two other patients (patients #2 and #23, Table 3) showed BRAF mutations with either NRAS or KRAS. Three patients showed multi-site mutations in the KRAS or BRAF gene (patients #6, #7, and #10, Table 3). However, no significant mutations were found in the 12 patients with AL amyloidosis alone, although mutations were found in myeloma with AL amyloidosis (Figure 1B). Although some patients showed more than 30% allele frequency, the majority of mutations in the three genes showed low variant allele fractions (around 5%). Mutations in BRAF and KRAS were verified using droplet digital PCR (ddPCR), and the values of allele frequency from the 7 patients harboring low allele fraction (≤ 10%) mutations were highly correlated with those of ddPCR (*r* = 0.95) (Figure 2). Thus, the application of a targeted gene sequencing panel in myeloma patients identified mutations in mitogen activated protein kinase (MAPK) pathway-related genes (KRAS, NRAS, and BRAF). Interestingly, the low allele frequency mutations were detected in separate sequence reads from those with high allele frequency mutations (Supplementary Figure S1), demonstrating the ongoing alteration of MAPK pathway for the wild type tumor populations. These data suggest that the high sensitivity of our panel sequencing platform could allow for early detection of subclones with clinical significance.

**Table 3:** Amino acid changes and allele frequencies of three genes (KRAS, NRAS, and BRAF) in co-occurrence samples

Sample	Amino acid change (variant allele frequency, %)		
	NRAS	KRAS	BRAF
	G12A(1)		
#1	G13R(14)	Q61R(3)	D594N(5)
	Q61K(2)		
#2	Q61L(18)		D594G(4)
#23		G12A(2)	D594N(7)
		A146V(5)	V600E(4)
#6		G12C(1)	
		G13D(28)	
#7		G13D(56)	
		L19F(3)	
#10			V600E(34)
			I714V(33)

### Copy number variations in myeloma and AL amyloidosis

**Figure 3:**
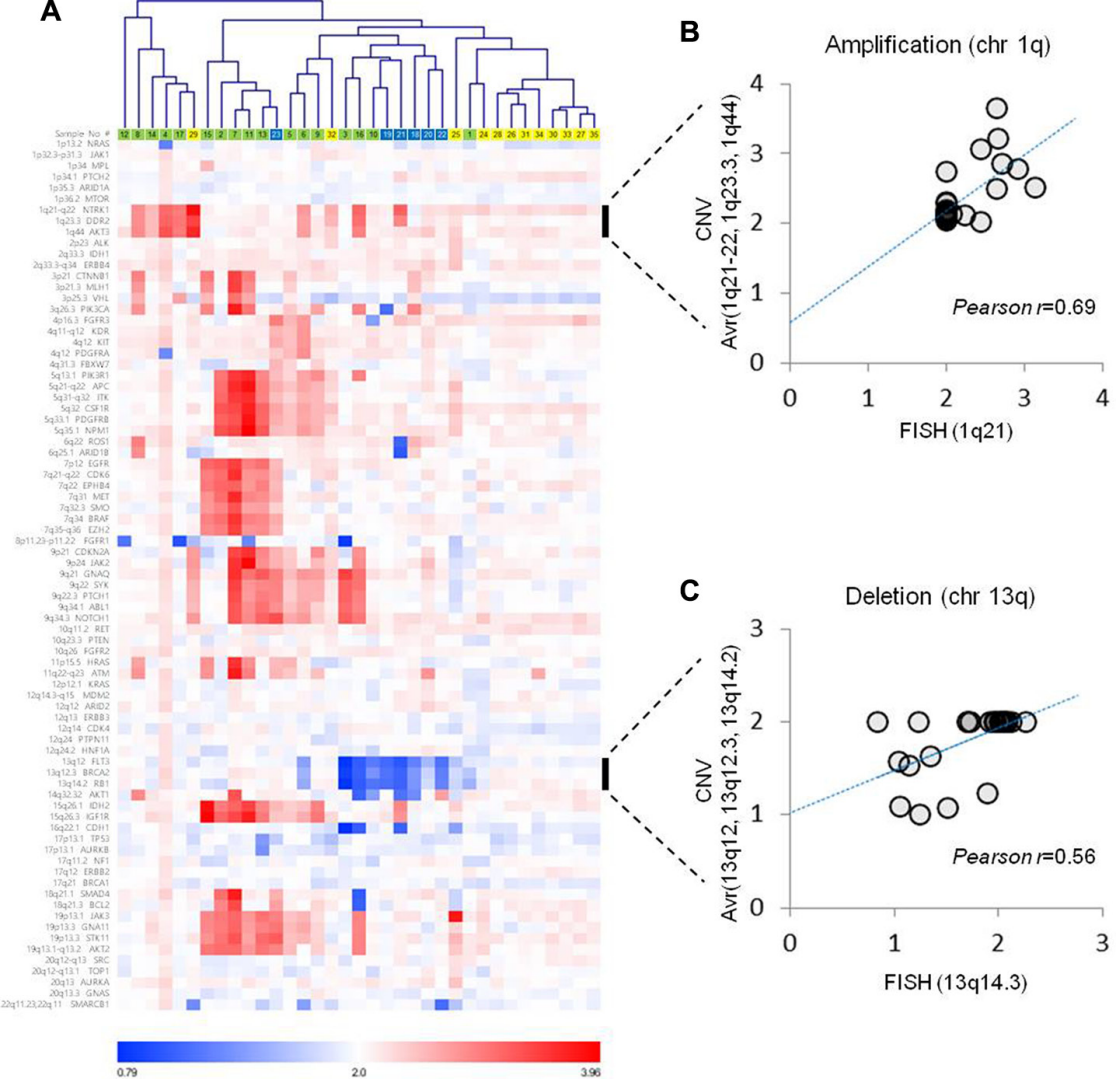
Copy number variations estimated from gene panel sequencing (**A**) Pearson's correlation distances were used for the average-linkage hierarchical clustering of patients. Correlation analysis between the CNV estimation and FISH analysis for (**B**) chromosome 1q amplification and (**C**) chromosome 13q deletion.

In addition to mutational analysis, gene panel sequencing was used to detect chromosomal copy number variations (CNV), which are often associated with prognosis in myeloma patients. Three types of CNV were detected in myeloma patients in a mutually exclusive manner, while amyloidosis patients had scarcely any changes (Figure 3A). The three CNVs in myeloma patients were amplification of chromosome 1q, amplification of odd number chromosomes (hyperdiploidy), and deletion of chromosome 13q. In particular, the chromosome 13q deletion was frequently detected in patients with the dual diagnosis of myeloma and amyloidosis (5 out of 6). Notably, chromosome 1q amplification and 13q deletion are associated with poor prognosis in multiple myeloma [15, 16]. The CNV calls made from gene panel sequencing showed a significant correlation with those from the whole exome sequencing (Supplementary Figure S2), and a lower positive correlation with those from fluorescence *in situ* hybridization (FISH) data using a single probe (Figure 3B, 3C).

### Association of treatment response and NRAS/KRAS/BRAF mutation

Among the seven patients with NRAS mutation, four patients (patients #1–4) were treated with bortezomib-containing treatment; three of these patients did not respond (Table 1). Thus, 75% (3/4) of patients showed disease progression during bortezomib treatment, although the sample size was small. Two patients with BRAF mutations also failed to show response to bortezomib; however, these patients (patients #1 and #2) both had NRAS mutations. Although only two patients with KRAS mutations received the bortezomib-containing treatment (patient #1, 8), neither responded to it. (One patient showed disease progression while the other maintained a stable disease state; Table 1). There was no significant association between the presence of the mutation and the response to other drugs such as thalidomide (data not shown).

## DISCUSSION

In this study, we identified mutations in three well-known oncogenes, KRAS, NRAS, and BRAF, in multiple myeloma patients. No mutations were found in patients with AL amyloidosis alone, although patients with myeloma combined with AL amyloidosis did show mutations in all three genes (Figure 1B). The frequency of the mutations in the three genes identified in our study was consistent with those presented in a recent study of whole-exome sequencing in 463 patients with myeloma enrolled in the National Cancer Research Institute Myeloma XI trial, which reported a dominant mutation in the RAS (43%) [17]. Mutations in RAS gene family have been reported to show predominant mutational activation of one member of the RAS gene family. Solid tumors such as colorectal and pancreatic cancers showed frequent KRAS mutations, whereas some hematologic cancers such as acute lymphoblastic leukemia predominately showed NRAS mutations [18]. Unlike other malignancies, mutations of KRAS and NRAS were found in approximately equal rates in myeloma [19, 20]. Similarly, the frequency of mutations was similar between NRAS (30%, 7/23) and KRAS (26%, 6/23) in our study (Figure 1B). BRAF mutations (22%, 5/23) were also found in five myeloma patients including two patients with both AL amyloidosis and myeloma (Figure 1B). The BRAF mutation is known to be mutually exclusive with KRAS and/or NRAS mutations in other cancers [21]; however, in our study, mutations in BRAF did not appear to be exclusive to either the KRAS or NRAS mutation because mutations in KRAS, NRAS, and BRAF were all found in the same patient (patient #1, Table 3). Given that each RAS family member can provide a similar oncogenic signal through the mitogen-activated protein kinase (MAPK) pathway promoting cell proliferation, the presence of these mutations could be expected to result in a worse treatment outcome than the absence of these mutations. Indeed, a previous study showed an association of NRAS mutations with a lower response rate to bortezomib and a shorter time to disease progression in bortezomib-treated patients [22]. In our study, patients with NRAS mutations showed poor response to bortezomib-containing treatments. Furthermore, the patient with mutation of three genes, KRAS, NRAS and BRAF (patient #1, Table 3) was primarily refractory to bortezomib, lenalidomide, and bendamustine-containing treatments. None of the mutations in the MAPK pathway-related genes were detected by conventional molecular tests at the time of initial diagnosis. Thus, a targeted sequencing such as our cancer panel may be useful for detecting low frequency genetic events, allowing for a predicted treatment response. As our patients with AL amyloidosis were mainly treated with thalidomide or alkylating agents, the association of the absence of the MAPK-pathway related gene mutations with the response to bortezomib could not be evaluated in this study (Table 2). However, our previous study with AL amyloidosis patients showed that sixteen of nineteen (84%) patients who were treated with bortezomib, melphalan, and prednisolone had a hematologic response, including seven complete responders [23]. These results support the association of MAPK pathway gene mutations with the response to bortezomib.

Our pilot study applied a targeted gene sequencing panel consisting of 83 genes that were selected based on the availability of corresponding targeting agents. Thus, the genes used for the targeted sequencing could be targeted by developed anti-cancer drugs. Furthermore, gene panel sequencing provided large numbers of mutations as well as chromosomal amplifications or deletions with an important prognostic value compared to the conventional cytogenetic and FISH methods. As there are discrepancies between gene panel sequencing and conventional cytogenetic/FISH methods, further improvement is required for the use of next generation sequencing for cytogenetic analysis.

Compared to myeloma and myeloma with AL amyloidosis, the results of AL amyloidosis showed no evidence of major mutations. Indeed, the clinical features and treatment outcomes were mainly correlated with the type and extent of organ involvement rather than by the number of plasma cells in patients with AL amyloidosis [24, 25]. By comparison, a previous study with longitudinal whole genome sequencing of a high-risk myeloma patients demonstrated tumor heterogeneity at diagnosis and identified potential mutations contributing to myeloma development as well as transformation from myeloma to overt extramedullary disease [26]. Accordingly, genetic alterations could accumulate during the process of myeloma development and progression contributing to genetic heterogeneity of myeloma. This heterogeneity also might influence treatment outcomes of myeloma because certain clones with a given genetic mutations may be sensitive to one drug whereas a different clone having with a different genetic mutations may not respond to the drug, resulting in relapse or progression after treatment. Thus, the initial screening of the genetic mutation landscape for each myeloma patient at diagnosis may provide important information for subsequent treatment and follow-up of myeloma patients.

Nonetheless, our study has some limitations. First, our gene panel mainly consisted of genes covering targets of developed anti-cancer drugs. Given that the majority of targeted agents currently available focus on solid tumors, a substantial number of genes studied were related to solid tumor phenotypes rather than to myeloma. It is also possible that some patients had alterations in non-target regions. Second, we could not demonstrate an association of mutations in MAPK pathway-related genes (KRAS, NRAS and BRAF) with survival outcomes of our patients. Thus, further study with larger study populations and longer follow-ups are required to confirm the clinical utility of targeted sequencing. Recently, Kortum et al. investigated 72 untreated myeloma patients with deletion 17p using a myeloma-specific gene panel for targeted sequencing of 47 genes. They found a higher prevalence of TP53 mutation (28%) than previous studies and suggested that this targeted sequencing could provide a comprehensive insight into the mutational landscape of high-risk myeloma [27].

In conclusion, we present results of our pilot study with targeted genome sequencing for myeloma and AL amyloidosis. We believe that targeted gene panel sequencing will help to further improve myeloma diagnosis as well as track clonal heterogeneity. Furthermore, this diagnostic approach may provide more precise prognosis and better guidance for treatment decisions in myeloma patients.

## MATERIALS AND METHODS

### Patients

We enrolled consecutive patients who were diagnosed with multiple myeloma or AL amyloidosis at the Samsung Medical Center between 2013 and 2014. Patients were diagnosed with myeloma according to the presence of malignant plasma cells ≥ 10% of bone marrow aspirates and increased monoclonal proteins in serum and/or urine [28]. AL amyloidosis was diagnosed with the presence of amyloid deposits in involved organs, which was confirmed by Congo red staining and monoclonal protein in serum and/or urine. After obtaining informed consent from participating patients, we performed bone marrow aspiration. Plasma cells were isolated by magnetic separation using anti-CD138 microbeads and were stored at −80°C. The extent of disease in myeloma patients was determined using the International Staging System (ISS) consisting of serum albumin and beta-2 microglobulin [29]. All patients with myeloma received induction treatment with curative intent. Induction treatment was done with immunomodulatory agents or proteasome inhibitors according to the physicians' discretion. After induction treatment, ASCT was done for eligible patients. To diagnose AL amyloidosis, patients receiving ASCT were < 65 years with adequate cardiac function. However, patients ineligible for ASCT received only an alkylating agent containing a chemotherapeutic agent such as melphalan or dexamethasone. The hematologic response to treatment was based on the revised uniform response criteria by the International Myeloma Working Group [30]. The survival and disease statuses were updated at the time of analysis; thus, the last update was done in March, 2016. All aspects of the study were reviewed and approved by the Institutional Review Board (IRB) of the Samsung Medical Center (No. 2012-08-059).

### Targeted sequencing

Targeted sequencing was done using a customized cancer panel. Genomic DNA was extracted from tissue specimens using QIAamp DNA mini kit (Qiagen, Valencia, CA, USA). Genomic DNA quality and quantity were determined using a Nanodrop 8000 UV-Vis spectrometer (Thermo Scientific, Waltham, MA, USA), a Qubit 2.0 Fluorometer (Life Technologies Inc., Grand Island, NY, USA), and a 2200 TapeStation Instrument (Agilent Technologies, Santa Clara, CA, USA). Genomic DNA (250 ng) from each tissue was sheared with Covaris S220 (Covaris, Woburn, MA, USA) and was used for the construction of a library using probes and the SureSelect XT reagent kit, HSQ (Agilent Technologies) according to the manufacturer's protocol. As previously reported, this panel is designed to enrich exons of 83 genes covering 366.2 kb of the human genome [31]. After the enriched exome libraries were multiplexed, the libraries were sequenced using the HiSeq 2500 sequencing platform (Illumina, San Diego, CA, USA). Briefly, a paired-end DNA sequencing library was prepared through gDNA shearing, end-repair, A-tailing, paired-end adaptor ligation, and amplification. After hybridization of the library with bait sequences for 27 hr, the captured library was purified and amplified with an index barcode tag, and the library's quality and quantity were measured. Sequencing of the exome library was carried out using the 100 bp paired-end mode of the TruSeq Rapid PE Cluster kit and the TruSeq Rapid SBS kit (Illumina).

### Variant detection

Sequence reads were mapped to the human genome (hg19) using the Burrows-Wheeler Aligner (BWA) [32]. Duplicate read removal was done using Picard (v1.93) and Samtools [33]. Local alignment was optimized using The Genome Analysis Toolkit (GATK) [34]. Variant calling was done only in targeted regions of the cancer panel. To detect single nucleotide variants, we integrated results of three kinds of variants caller [35–37], which increased sensitivity. We used a Pindel to detect insertions and deletions [38]. Copy number variations were calculated for targeted regions by dividing read-depth per exon by the estimated normal reads per exon using an in-house reference. Gene fusions in the target region were identified using an in-house algorithm.

### Digital droplet PCR

Digital droplet PCR was performed on a QX200 ddPCR™ System (Bio-Rad, Hercules, CA, USA) platform. Briefly, ddPCR™ reaction mixes were prepared with template gDNAs, ddPCR™ Supermix (Bio-Rad), and TaqMan primer-probe mixtures and were partitioned into oil droplets (∼20,000) generated by a QX200 droplet generator. The droplets were then thermal-cycled under the same condition described above using Veriti 96-Well Thermal Cycler (Life Technologies). Amplified droplets were imaged on a QX200 droplet reader (Bio-Rad) and analyzed by QuantaSoft™ software (Bio-Rad). The concentration of nucleic acid sequence targeted by the FAM and VIC or FAM and HEX dye labeled probes was estimated by Poisson distribution.

### Statistical analysis

The Kaplan–Meier method was used for univariate analysis of survival outcomes. Survival outcomes were compared with the log-rank test. Overall survival was measured from the date of diagnosis to the date of death due to any cause and was censored at the date of the last follow-up visit. A two-sided *P value* < 0.05 was considered statistically significant.

## SUPPLEMENTARY MATERIALS FIGURES AND TABLE


