# Supplementary material for: Recurrent mutations of MAPK pathway genes in multiple myeloma but not in amyloid light-chain amyloidosis
Source: Oncotarget. 2016 Sep 15;7(42):68350–9. doi: 10.18632/oncotarget.12029 (PMC5356560; doi:10.18632/oncotarget.12029)
Supplement: Supplementary file 1 [file oncotarget-07-68350-s001.pdf]

Recurrent mutations of MAPK pathway genes in multiple myeloma but not in amyloid light-chain amyloidosis

Supplementary Materials

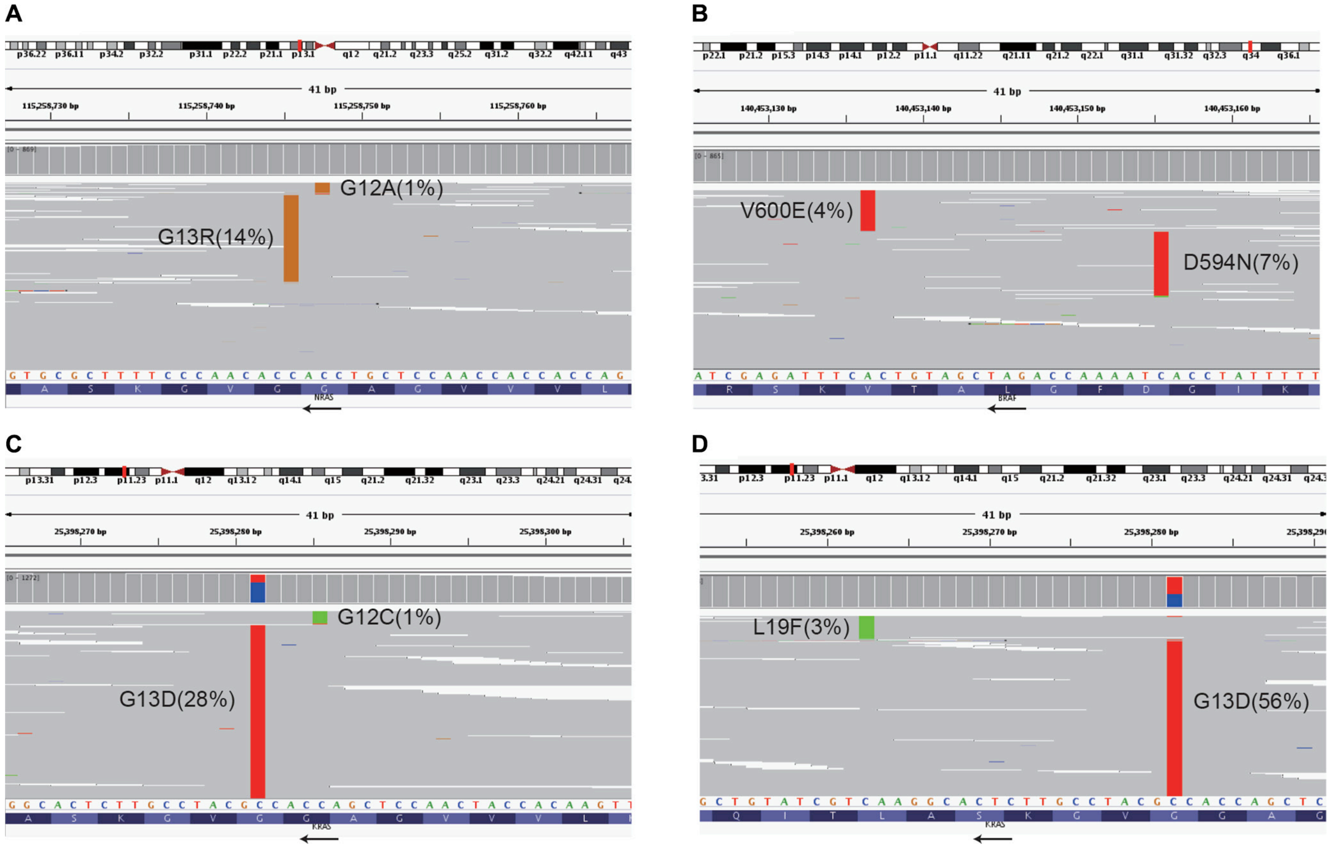

Supplementary Figure S1: Mutations on a same gene are located in *trans* alleles. (**A**) *NRAS* G13R(14%) and G12A(1%) of sample 1. (**B**) *BRAF* V600E(4%) and D594N(7%) of sample 23. (**C**) *KRAS* G13D(28%) and G12C(1%) of sample 6. (**D**) *KRAS* L19F(3%) and G13D(56%) of sample 7. Arrows indicate a strand of the gene.

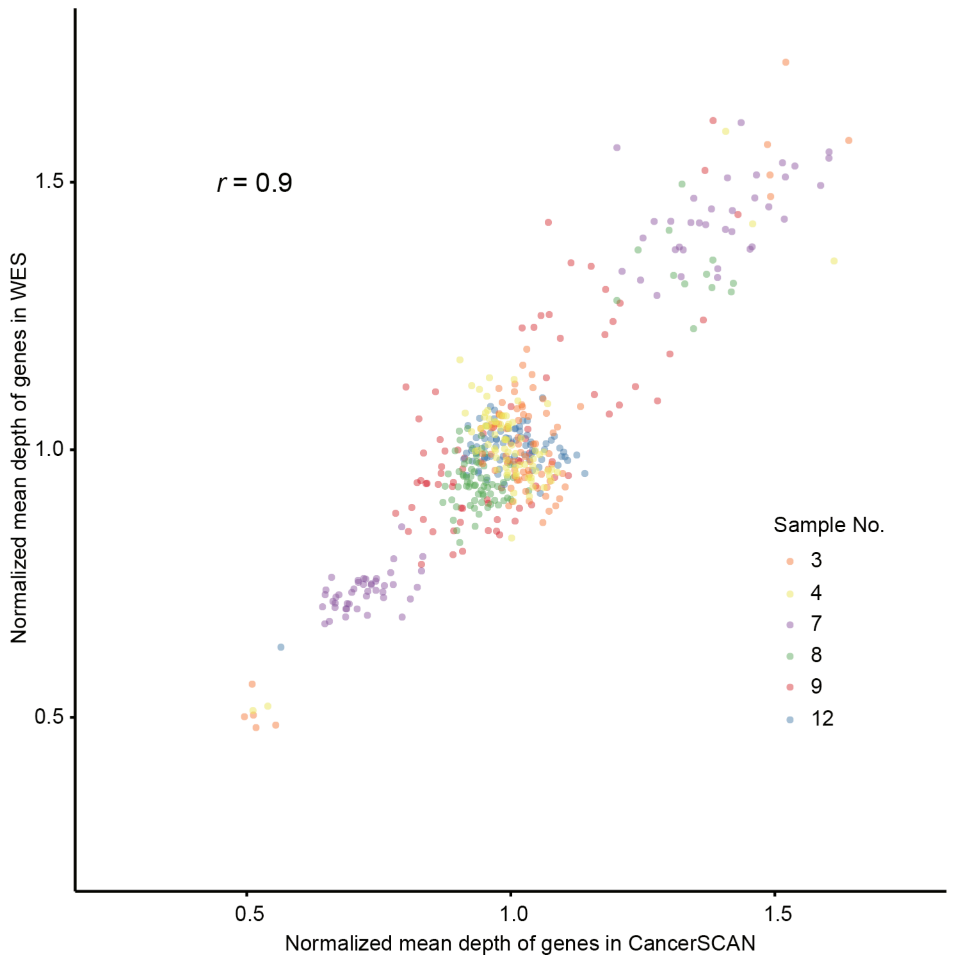

Supplementary Figure S2: Correlation of mean depth of target genes between CancerSCAN and whole exome sequencing (WES) of 6 samples. Libraries of WES was constructed by SureSelect XT Human All Exon v5 and the libraries were sequenced on HiSeq 2500. Depth of WES and CancerSCAN were normalized using pair normal and in-house reference, respectively. Among target genes, genes for only fusions and promoter and on X chromosome were excluded because normlization of the genes is infeasible.

Supplementary Table S1: Sequencing statistics of targeted sequencing

No.	GC (%)	Q30 (%)	Duplication (%)	Coverage	≥ 100× (%)
1	45	89.8	30.06	696.4	99.0%
2	41.5	91.7	7.99	304.5	97.1%
3	45	91.2	13.06	819.6	98.9%
4	45	90	15.61	928.8	99.1%
5	42	91.7	12.55	639.7	98.8%
6	45	89.6	24.78	1048.4	99.2%
7	45	91.3	12.71	968.9	98.9%
8	45	90.7	11.9	1003.7	99.1%
9	45	91.5	18.23	1101.5	99.2%
10	45	90.8	16.53	1153.8	99.2%
11	44	90.7	17.77	1118.4	99.1%
12	44	90	25.58	1048.8	99.2%
13	45	90.9	17.67	1120.3	99.2%
14	45	89.9	12.48	971.7	99.1%
15	45	90.5	12.95	929.4	98.9%
16	43	91	11.42	606.6	98.7%
17	45	91.1	18.5	801.1	99.0%
18	45	92.7	9.01	773.7	99.1%
19	45	90.7	13.19	1021	99.1%
20	45	91.2	7.96	697.5	98.9%
21	46	92.7	8.22	812.2	98.9%
22	45.5	92.9	8.1	787	99.1%
23	46	93.2	11.67	789.8	99.0%
24	45	90.3	30.39	893.5	99.1%
25	45.5	88.4	9.24	849.9	99.1%
26	45	92.6	8.36	838.2	99.1%
27	46	92.4	10.13	698	99.1%
28	45	92.5	7.59	822.2	99.0%
29	45.5	84.9	9.79	798.3	99.0%
30	45	85.2	15.32	707.8	99.1%
31	45.5	87.7	9.44	770.3	99.0%
32	45.5	88.7	8.48	778.7	99.0%
33	45.5	90.9	9.84	821.3	99.1%
34	45	90.9	12.06	748.6	99.1%
35	45.5	89.6	8.85	747.5	99.1%
Mean	44.9	90.6	13.6	846.2	99.0%
